# Cytotoxic Pathways in Allogeneic Hematopoietic Cell Transplantation

**DOI:** 10.3389/fimmu.2018.02979

**Published:** 2018-12-19

**Authors:** Wei Du, Xuefang Cao

**Affiliations:** ^1^Department of Immunology, Roswell Park Comprehensive Cancer Center, Buffalo, NY, United States; ^2^Department of Microbiology and Immunology, Marlene and Stewart Greenebaum Comprehensive Cancer Center, University of Maryland, Baltimore, MD, United States

**Keywords:** allogeneic hematopoietic cell transplantation (allo-HCT), graft-vs.-host disease (GVHD), graft-vs.-tumor (GVT) effect, cytotoxic pathways, the Fas/Fas ligand (FasL) system, the perforin/granzyme pathway, cytokines

## Abstract

Allogeneic hematopoietic cell transplantation (allo-HCT) is a potentially curative treatment for hematologic malignancies, and other hematologic and immunologic diseases. Donor-derived immune cells identify and attack cancer cells in the patient producing a unique graft-vs.-tumor (GVT) effect. This beneficial response renders allo-HCT one of the most effective forms of tumor immunotherapy. However, alloreactive donor T cells can damage normal host cells thereby causing graft-vs.-host disease (GVHD), which results in substantial morbidity and mortality. To date, GVHD remains as the major obstacle for more successful application of allo-HCT. Of special significance in this context are a number of cytotoxic pathways that are involved in GVHD and GVT response as well as donor cell engraftment. In this review, we summarize progress in the investigation of these cytotoxic pathways, including Fas/Fas ligand (FasL), perforin/granzyme, and cytokine pathways. Many studies have delineated their distinct operating mechanisms and how they are involved in the complex cellular interactions amongst donor, host, tumor, and infectious pathogens. Driven by progressing elucidation of their contributions in immune reconstitution and regulation, various interventional strategies targeting these pathways have entered translational stages with aims to improve the effectiveness of allo-HCT.

## Introduction

Allogeneic hematopoietic cell transplantation (allo-HCT) is a potentially curative treatment for leukemia, lymphoma, and other hematologic malignancies. It is also an effective therapy for some non-malignant diseases, such as aplastic anemia, immunodeficiencies, and autoimmune diseases (1, 2). In allo-HCT, donors and recipients must have at least partially matched human leukocyte antigen (HLA) genotype to ensure engraftment and decrease the possibility and severity of graft-vs.-host disease (GVHD) (3). After the recipients are treated with conditioning regimens that include high-dose chemotherapy or combined with radiotherapy, donor bone marrow cells or peripheral blood stem cell (PBSCs) mobilized by granulocyte colony-stimulating factor (G-CSF) are infused to the recipients. The cells in the allogeneic graft, which include hematological stem cells and pre-existing immune cells, are not only important for re-establishing the hematological system, but also critical for reconstitution of immunity against tumor and infectious pathogens (4). In case of malignant diseases, donor immune cells are able to attack and eradicate residue malignant cells. This unique immune response has been defined as the graft-vs.-tumor (GVT) effect (5). However, the development of GVHD may limit the success of allo-HCT, which results from donor allogeneic T cells damaging normal recipient tissues as foreign (4, 6, 7). Acute GVHD may develop within a few weeks after allo-HCT, characterized by damage to susceptible organs, causing skin lesion, liver dysfunction, and diarrhea. Chronic GVHD occurs later leading to further damage to connective tissue, respiratory tract, and exocrine glands. Multiple modalities, including T cell depletion (TCD), immunosuppressive agents and different conditioning regimens, have being utilized to prevent or treat GVHD. Nevertheless, these strategies are not always effective, and may adversely cause infection, cancer relapse, or secondary malignancies (4, 6). Therefore, the “holy grail” of allo-HCT remains the separation of the adverse GVHD from the desired GVT effect.

It has been established that many types of donor-derived immune cells, such as different subsets of T cells (4, 8), B cells (9, 10), and NK cells (11, 12) are involved in mediating GVHD and GVT effect. Donor-derived T cells remain the main player for both GVHD and GVT response. Simply depleting T cells from the allo-graft could successfully prevent GVHD (13), but increases the risk of cancer relapse (14). Most of the therapeutic approaches for GVHD are targeting T cells, such as T cell modulation in different stages of transplantation (15, 16), co-stimulatory and co-inhibitory modulation (17–21), and targeting cytokines produced by T cells (22–24). The most practiced GVHD therapy still use glucocorticoids that have strong and broad anti-inflammatory effects including suppression of T cell-mediated cytotoxicity (25).

**Figure 1:**
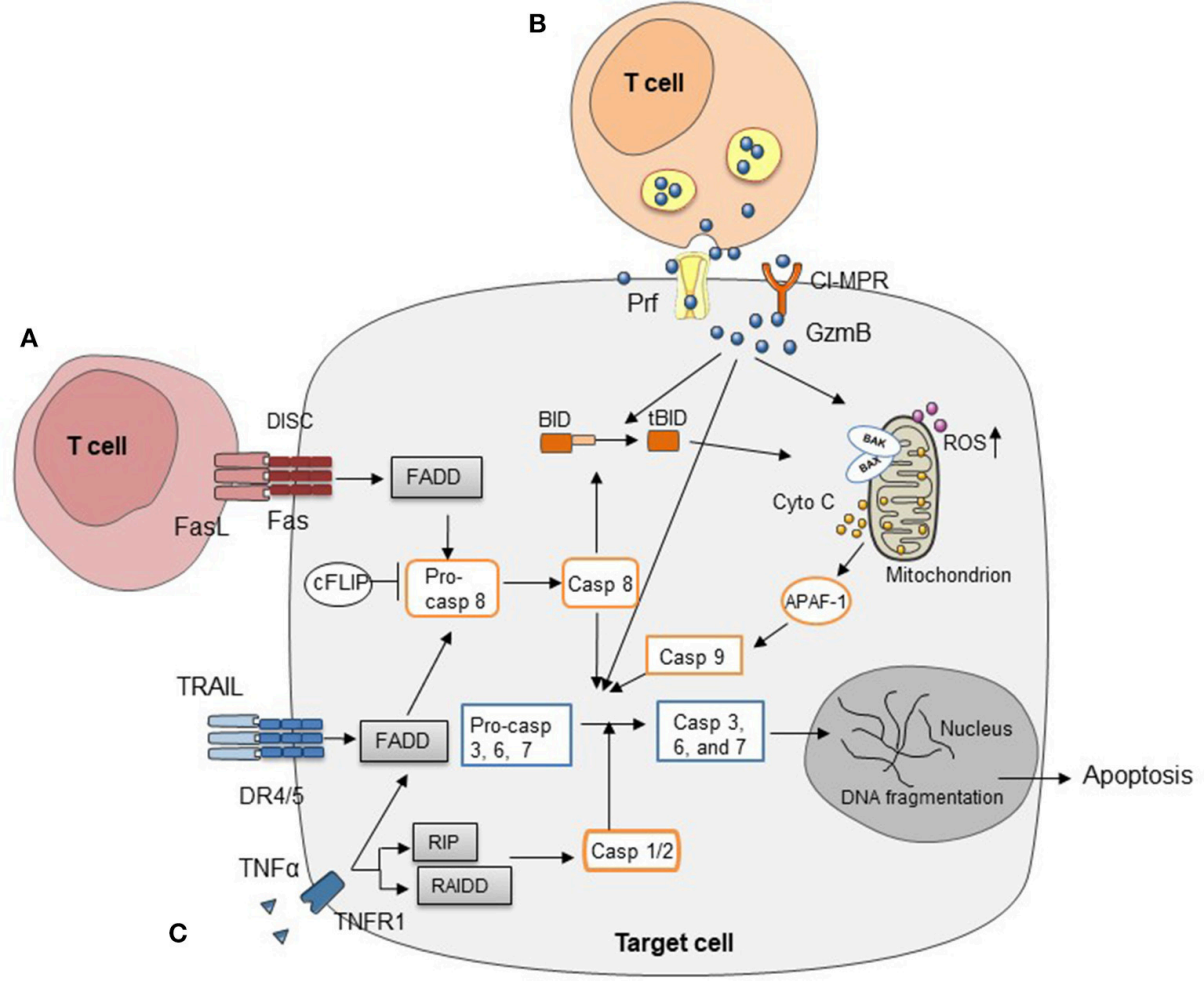
Three major cytotoxic pathways in HCT **(A)** FasL on T cells induces target cell apoptosis by engaging Fas on cell surface. **(B)** Cell apoptosis mediated by perforin/granzymes stored in the cytotoxic granules of T cells. **(C)** Cytokines secreted by T cells, such as TNFα, IFNγ, and TRAIL, mediate target cell apoptosis through various signaling pathways.

Both CD4^+^ and CD8^+^ T cells can cause GVHD (26). At the molecular level, a number of pathways have been described for allogeneic T cell-mediated cytotoxicity, including Fas/Fas ligand (FasL), perforin/granzymes, and cytokines such as tumor necrosis factor α (TNFα), interferon γ (IFNγ), and TNF-related apoptosis-inducing ligand (TRAIL) (27–29) (Figure 1). Many studies have examined these pathways in allo-HCT. Interestingly, most of these T cell-derived cytotoxic molecules can affect both target cells and T cells themselves, while different T cell subsets (e.g., CTLs vs. Tregs) can use the same molecule to perform distinct functions thereby causing different impact on GVHD and GVT response (28, 30–32). In principle, the Fas/FasL pathway has been reported to function mainly in CD4^+^ T cell-mediated GVHD, while the perforin/granzyme pathway is essential in CD8^+^ T cell-mediated GVHD (33). In addition, many reports have demonstrated the importance of cytokines in regulating GVHD and GVT effects (34–39). In this review, we provide updates for research progress and treatment strategies targeting these cytotoxic pathways.

## Fas/FasL Pathway in Allo-HCT

Fas, also known as CD95, belongs to TNF receptor superfamily and is expressed in multiple organs, playing a crucial role in extrinsic programmed cell death. FasL, also known as TNFL6, is predominantly expressed on activated T cells, macrophage, and neutrophils. Fas is a type I transmembrane receptor protein, existing as a homotrimer. Once engaged by FasL, Fas will trigger the formation of the death-inducing signaling complex (DISC). Subsequently, Fas interacts with the adaptor protein Fas-associated death domain protein (FADD) through homologous domain (40). This triggers the autocatalytic cleavage of pro-caspase 8 into caspase 8 and activation of downstream molecules, such as caspase 3, caspase 6, and caspase 7, which eventually induce apoptosis. Caspase 8 can activate the mitochondrial cell death pathway as well, resulting in activation of cytochrome c and caspase 9 (40).

Fas or FasL deficiency in mice (Fas receptor mutation *lpr* mice and FasL deficiency *gld* mice) causes accumulation of TCRαβ^+^CD3^+^B220^+^CD4^−^CD8^−^ double negative (DN) T cells and systemic lupus erythematosus like autoimmune disease, which indicated Fas/FasL pathway plays an important role in T cell negative selection in thymus (41, 42). Fas mutation in human can also cause autoimmune lymphoproliferative syndrome (ALPS) (43). Activation-induced cell death (AICD), defined as activated T cells undergoing apoptosis after ligation of TCR by antigen or mitogen, has critical regulatory function of T cell response. Fas/FasL pathway is essential for AICD of T cells, T cell selection during development, as well as mature T cell re-stimulation by antigens (44, 45).

### Fas/FasL in GVHD

Increased expression of Fas and FasL is observed in both CD8^+^ and CD4^+^ T cells during GVHD (46–48) and is associated with the severity of GVHD (48, 49). Blockade of Fas/FasL pathway led to decreased overall mortality in GVHD (50, 51) and reduced tissue specific organ damage (52). Meanwhile, single-nucleotide polymorphism (SNP) analysis showed that SNP of Fas in recipients can be used to improve prognostic stratification of GVHD (53, 54). Furthermore, selective depletion of host-sensitized donor lymphocytes by pre-treatment of soluble FasL can prevent GVHD (54–56). These results indicate that Fas/FasL is a key molecule in the pathogenesis of GVHD. Mizrahi et al. (57) found that short-term mobilization of peripheral blood by FasL reduced GVHD and improved survival following lipopolysaccharide stimulation, while retaining GVT activity. Likewise, engineered T cells displaying novel form of FasL (streptavidin-FasL) eliminated alloreactive T cells without significantly affecting GVT effect (58). However, the expression level of Fas failed to serve as a sensitive and specific marker for GVHD (59).

Variable mechanisms have been proposed for the function of Fas/FasL pathway in GVHD. Using murine parent to F1 models, it was reported that FasL pathway was important for both CD4^+^ and CD8^+^ T cell-mediated GVHD. Host mice receiving FasL-deficient donor T cells developed significantly less GVHD compared with WT donor T cells (60). FasL-deficiency in donor T cell did not affect T cell proliferation, homing, activation, cytokine production, and anti-tumor activity, but decreased mature T cell expansion after allo-HCT (50, 60). However, allo-HCT of FasL-deficient T cells led to decreased donor cell engraftment and subsequent chimerism (61). On the recipient side, both Fas-deficient and FasL-deficient mice had higher GVHD mortality compared with WT mice (62, 63). Together, these findings show that Fas/FasL pathway in the host is vital to resist donor cell engraftment and subsequent GVHD, while important for donor cell engraftment in allogeneic host to form stable chimerism after non-myeloablative conditioning. Therefore, how to attenuate Fas-mediated GVHD, while not affecting donor cell engraftment is a great challenge. Further study showed brief exposure of unstimulated naïve donor lymphocytes to FasL *in vitro* preferentially depleted FasL-sensitive cells, and attenuated GVHD without impairing engraftment or GVT activity (64). In addition, FasL had been found to enhance the killing activity of CD25^+^ regulatory T cells (killer Treg) and abrogate autoimmunity. Infusion of killer Treg cells increased apoptosis of effector lymphocytes and ameliorated GVHD severity (65).

Previously, it was believed that CD4^+^ T cells cause cytotoxicity mainly through Fas/FasL pathway while CD8^+^ T cells prefer the perforin/granzyme pathway (66). However, reports afterwards demonstrated that the perforn/granzyme pathway was involved in cytotoxic function of CD4^+^ T cells and Fas/FasL is important for that of CD8^+^ T cells as well, though the potency was variable (60, 67). Maeda et al. (68) reported that deficiency in either perforin or FasL in CD8^+^ T cells decreased the development of GVHD, indicating that both were required for the function of alloreactive CD8^+^ T cells. However, another study showed that donor T cell cytotoxicity via Fas/FasL or perforin was not prerequisite for induction of GVHD (69). T cells lacking perforin and FasL function can still cause lethal GVHD after bone marrow transplantation (69). Furthermore, it was reported that memory CD8^+^ T cells in the host mediated resistance to donor cell engraftment through a mechanism that was independent of FasL and perforin pathways (70). Sleater et al. (71) demonstrated that the absence of either perforin or Fas had little impact on rejection of pancreatic islet. However, simultaneous disruption of both pathways prevented allograft rejection despite T cell infiltration. These findings painted a complicated picture about how Fas/FasL in the host and donor cells affect GVHD. We postulate that the perforin/granzyme and Fas/FasL pathways comprise alternative and required mechanisms for T cell-mediated cytotoxic function in the context of allo-HCT. In addition, FasL is the critical for NK cell-mediated cytotoxicity. Donor NK cells have been found to suppress GVHD while inducing GVT effect after allo-HCT (72, 73). Olson et al. also showed that co-injection of donor NK cells with alloreactive T cells decreased host GVHD severity by reducing cytokine production, T cell activation, and proliferation, via a mechanism that involved T cell apoptosis induced by NK cells through the FasL and perforin pathways (74).

### FasL/Fas in Target Organ Damage

Skin, liver, and intestines are typical target organs in acute GVHD, while primary and second lymphoid organs are also susceptible. In a human skin explant model, higher GVHD score was associated with Fas expression in epithelium and blockade of Fas-mediated apoptosis decreased severity of cutaneous GVHD damage (75). Likewise, in oral mucosa lesions, allogeneic lymphocytes from FasL-defective mice did not induce vascular damage, or epithelial cell death in recipients, suggesting a major role of FasL by allogeneic lymphocyte-mediated mucosal GVHD (76). It was found that radiation conditioning prior to allo-HCT upregulated Fas expression on thymic stromal cells and donor alloreactive T cells used FasL to medicate thymic GVHD (77). In addition, bone marrow atrophy is mediated by p53-dependent up-regulation of Fas (78). Ceramide-rich macrodomains are sites where Fas is concentrated on cells. Sphingomyelinase-deficient mice, which cannot generate ceramide, revealed reduced GVHD-related organ damage, attenuated cytokine storm, and CD8^+^ T cell proliferation. These results indicate that GVHD-mediated cutaneous damage is associated with Fas expression in recipients (79). However, studies of GVHD in liver and intestines are controversial. Hepatotoxicity is more likely through FasL-Fas pathway (80), while intestinal GVHD is associated with FasL-dependent TNFα level (81). Specifically, hepatic lesions were improved by administration of anti-FasL antibody whereas intestinal lesions were protected by anti-TNFα antibody but not by anti-FasL antibody (82). This result indicates that FasL and TNFα differentially contribute to GVHD pathogenesis. Contradictory results were also reported that administration of anti-FasL and anti-TNFα antibodies or using FasL-deficient donor T cells was not able to prevent intestinal GVHD (52, 82, 83). Furthermore, hematopoietic stem cells are also susceptible to FasL-induced cell apoptosis. A recent murine model study indicated that bone marrow cells pretreated with IFNγ increased expression of Fas and related caspases and proapoptotic genes which cause engraftment failure after allo-HCT (84). Therefore, it becomes evident that multiple pathways are involved in this sophisticated network and further investigations need to evaluate the role of Fas/FasL pathway in crosstalk with other molecules during GVHD target organ damage.

### FasL/Fas System in GVT Effect

Initially, the FasL/Fas system was believed to engage in GVHD only (32). Depletion of FasL led to decreased lethal GVHD while GVT activity remained intact, suggesting that other molecular pathways are responsible for GVT effect (85). However, another report showed that CD8^+^ T cell-mediated GVT activity depended on IFNγ and FasL but did not require TNFα, perforin or TRAIL (35). Other studies showed that FasL and perforin were both required for CD8^+^ T cell-mediated GVT effect (86), while the perforin/granzyme pathway may be more dominant in GVT activity mediated by CD8^+^ T cells (60). It is also possible that CD4^+^ T cell-mediated GVT effect relies on both the Fas/FasL and the perforin/granzyme pathways (60). These studies used different donor-host combinations for allo-HCT and various tumor models, which may explain the discrepancy on the contributions of these pathways to GVT effect. While there is not a comprehensive all-in-one model to quantify the contributions of various pathways to GVT activity in different cancers that are enormously heterogenous, it is reasonable to conclude that the FasL/Fas system is important for GVT effect against certain tumors.

## Perforin/Granzyme Pathway in Allo-HCT, GVHD, and GVT Effect

More than 30 years ago, a pore-forming molecule was observed on cells that were targeted by NK cells (87), which was later isolated, purified, and named perforin (88). A few years later, Jürg Tschopp's group purified a family of serine protease stored in cytoplasmic granules in cytotoxic T lymphocytes (CTLs) called granzymes (89). To date, five different granzymes have been identified in humans, named A, B, H, K, and M; while for mice, there are 10 functional granzymes, A, B, C, D, E, F, G, K, M, and N (90). Granzymes and perforin are packaged in CTLs and natural killer (NK) cells. When the killer cells engage their target cells, these proteins are released into the target cell membrane through synaptic cleft, where perforin mediates the influx of granzymes through forming pores on target cell membrane. Subsequently, granzymes cleave substrate proteins carrying out multiple functions. GzmA and B are expressed in CTLs and NK cells, whereas GzmK is expressed mainly in NK cells (91, 92). Different granzymes have various substrates specificity. GzmA and K exhibit tryptase-like activity and cleave substrates after arginine or lysine, whereas GzmB cleaves its target proteins after aspartic acid or glutamic acid. Among all these granzymes, GzmB, which is responsible for apoptosis, is the most extensively studied (93). Clipped by GzmB, pro-apoptotic BH3-only protein BID translocated to mitochondria causing cytochrome C release. Besides activation of mitochondria-mediated apoptosis, GzmB can directly process caspase-dependent pathways, including the effector caspase 3 and initiator caspase 8. A recent study also reported that GzmB directly attacked mitochondria and triggered increased production of reactive oxygen species (ROS) in target cells that was involved in causing apoptosis (94). To date, GzmB has been implicated in autoimmune disease, infection, cancer, and GVHD (95).

It was reported in 1996 that perforin was involved in the kinetics of GVHD induced by allogeneic T cells (52). Graubert et al. (33, 96) further showed that the cytotoxic effect of GzmB was pivotal for GVHD mediated by CD8^+^ T cells, but not by CD4^+^ T cells, and restricted in MHC I-mismatched GVHD. Then the contribution of this pathway to the GVT response was examined a few years later. Tsukada et al. (38) used mouse leukemia models to show that perforin-deficient donor cells lost GVT activity, leading to early death of the hosts due to leukemia outgrowth. On the other hand, a recent report showed that perforin-dependent CD8^+^ T cell apoptosis after donor lymphocyte infusion (DLI) impaired T cell proliferation and limited vaccine-based GVT effect (97). In addition, a recent published study from Galleu et al. (98) stated that mesenchymal stromal cells (MSCs) could be induced to undergo apoptosis in a perforin-dependent manner, which was essential to initiate MSC-induced immunosuppression after infusion to GVHD patients. Moreover, the cytotoxic activity delivered by either host CD8^+^ T cells or host CD56^+^ NK cells was correlated with less severe GVHD for patients who get MSC therapy. Therefore, the contribution of the perforin/granzyme pathway to GVHD and GVT effect is more complicated than initially believed and is involved in multiple aspects of GVHD.

### Perforin/Granzyme Pathway in T Regulatory Cells

Grossman et al. first showed that human adaptive Treg cells (converted from CD4^+^ conventional T cells) preferentially express GzmB and can kill allogeneic target cells in a perforin-dependent manner (99). On the other hand, human CD4^+^CD25^+^ natural Treg cells express GzmA but very little GzmB. Both Treg subtypes display perforin-dependent cytotoxicity against autologous target cells, including activated CD4^+^ and CD8^+^ T cells, CD14^+^ monocytes, and both immature and mature dendritic cells (100). Based on *in vitro* activation of human T cells, these findings suggest that the perforin/granzyme pathway is one of the mechanisms that human Treg cells use to control immune responses. A recent study from Choi et al. (101) reported that the hypomethylating agent azacytidine could drive Foxp3 expression in non-Treg cells and convert them into Tregs that could suppress GVHD without decreasing GVT effect in a murine model. And the suppressive function in those converted Tregs was partially dependent on perforin, but not GzmB. However, our studies with *in vivo* mouse tumor models showed that GzmB is important for natural Treg cell-mediated suppression of anti-tumor response (102). For natural Treg cell-mediated allogeneic T cell response, it was learned that GzmB was not required for donor natural Treg cell-mediated suppression of murine GVHD (103). Furthermore, our recent work has proven that GzmB is not required for natural Treg cell-mediated suppression of GVT effect either (104). Therefore, it seems that inhibiting GzmB will cause minimal influence on natural Treg-mediated suppression of murine GVHD and GVT effect. However, GzmA has recently been reported to be required for Treg-mediated suppression of murine GVHD, providing protection against GI tract damage (105). In a recent clinical study, Ukena S et al. analyzed CD4^+^CD25^hi^CD127^lo^ Treg population from patients with and without GVHD after allo-HCT and found that higher GzmA expression in Treg cells had better tolerance to allo-graft (106).

### Perforin/Granzyme Pathway in CD8^+^ and CD4^+^CD25^−^ Conventional T Cells

Using MHC I-mismatched and MHC-fully mismatched murine models, Graubert et al. reported in 1996 that GzmB was important for CD8^+^ T cells to cause lethal GVHD. GzmB deficiency in CD8^+^ T cells significantly decreased the lethality and severity of GVHD after transplantation (96). Recent studies by our lab added to two new discoveries. First, while GzmB^−/−^ CD8^+^ T cells exhibit reduced ability to cause GVHD, which was expected, surprisingly GzmB^−/−^ CD8^+^ T cells showed significantly enhanced GVT activity with several tumor models (107). GzmB-mediated activation-induced T cell death may account for the different anti-tumor immune responses between WT and GzmB^−/−^ CD8^+^ T cells. Secondly, we have found that a TLR5 agonist, could not only enhance GVT activity via activating antigen presenting cells (APCs) (108), but also stimulate up-regulation of endogenous GzmB inhibitor, Spi6, in accessory immune cells including APCs. In addition, our new report showed that Spi6 protects alloreactive T cells from GzmB-mediated mitochondrial damage, preserving their ability to cause GVHD without affecting GVT effect (109). Yet our study also suggests a novel function for Spi6, which contributes to GzmB-independent protection of intestinal epithelial cells in murine GVHD (110).

Initially, it was thought that GzmB was not important for CD4^+^ T cell-mediated GVHD. However, from our study published recently, we found that GzmB expression was upregulated in CD4^+^CD25^−^ conventional T cells after allo-HCT (111). GzmB^−/−^ CD4^+^CD25^−^ T cells exhibited enhanced expansion which was due to decreased activation-induced cell death (AICD). More GI tract damage and more cytokine production were observed in the hosts mice receiving GzmB^−/−^ CD4^+^CD25^−^ T cells. Using both MHC-mismatched (B6 to BALB/c) and minor antigen mismatched (129/SvJ to B6) models, we confirmed that GzmB^−/−^ CD4^+^CD25^−^ T cells caused more severe GVHD compared to WT counterparts (111). On the flip side, GzmB^−/−^ CD4^+^CD25^−^ conventional T cells partially lost GVT effect compared with WT T cells (104).

These new results reveal a more complicated paradigm for this pathway in allo-HCT as GzmB function in different T cell subsets (CD4^+^ vs. CD8^+^) unexpectedly leads to opposite outcomes in GVHD and GVT effect. Therefore, simply targeting GzmB in total T cell population is probably not beneficial for improving allo-HCT. Instead, disabling GzmB function in selected CD8^+^ donor T cells but not in CD4^+^CD25^−^ donor T cells may lead to favorable outcomes desired for allo-HCT patients.

## Other Cytotoxic Pathways in allo-HCT

Cytokines and their receptors are involved in different stages of GVHD, from T cell activation, differentiation, trafficking to direct tissue injury. In completed clinical trials, blockade of single cytokines alone, such as TNFα or interleukin-1 (IL-1), failed to improve clinical outcomes (24, 112) although there were evidence showing significant correlation between IL-1, IL-1β, and TNF-α and GVHD occurrence (113). The reason for these results is elusive and related to insufficient insight of complexity of the cytokine network. Latest advances in immunology and novel therapeutic agents suggest that the strategy of targeting cytokines needs to be revisited and may provide salutary effects on GVHD and GVT management.

### TNFα

TNFα is a type II transmembrane protein which can be cleaved to a soluble form. It has been well-studied and known as a pro-inflammatory cytokine (114, 115). Holler et al. first reported that increased blood level of TNFα was observed in acute GVHD (116). Soluble TNF receptors (TNFRs) were also associated with GVHD related complications (117). Choi et al. demonstrated the dynamical change of TNFR1 level before and after allo-HCT and its correlation with high grade II-IV GVHD (118). Clinical trial investigating the combination of TNFα monoclonal antibody (Etanercept) plus methylprednisolone as initial therapy for GVHD found substantial majority of remission, delayed onset of acute GVHD and reduced organ damage (112). Subsequent phase II trial revealed that lower TNFR1 level was linked with better prognosis. However, adding Etanercept to standard prophylaxis did not affect the overall rate of GVHD (119). In a multicenter prospective study, Etanercept arm had lower rate of GVHD initially, but achieved similar response in the end (120). Infliximab, a murine-human chimerized monoclonal antibody against TNFα, failed to lower the risk of GVHD in a small prospective study (121). In addition, TNFα has been a promising target in GVHD prevention particularly in gastrointestinal system (122). It should be noted that none of the studies stratify the patients based on their TNFα or TNFRs levels and these ambiguous findings should prompt us to revamp the conventional concept of TNFα. On the flip side, TNFα performs critical regulatory function in Treg cells after allo-HCT (123). In a murine allo-HCT study, donor Treg cells primed by TNFα can decrease GVHD, prolong animal survival and maintain GVT effect (124). Overall speaking, TNFα plays a fundamental role in allo-HCT, including GVHD initiation and progression, affecting clinical outcome and response to therapy, yet it functions much more like a pro-inflammatory cytokine than a cytotoxic molecule.

### IFNγ

IFNγ plays a central role in host defense by regulating both innate and adaptive immunity, including specific effects on T cell differentiation and proliferation (125). IFNγ exerts paradoxical effect in GVHD. Exogenous IL-12 treatment stimulates IFNγ-mediated protection against GVHD after lethal irradiation conditioning on the day of allo-HCT (126). However, using IFNγ knockout mice, two groups independently reported that neither donor nor host derived IFNγ is required for the development of GVHD (127, 128). Further studies confirm that the protective effect of IFNγ may depend on IL-12, IL-18, or Fas (129–131). Although the exact mechanism of IFNγ in GVHD remains unclear, it may implicate that IFNγ signaling in recipient non-hematopoietic cells is more important in the process of GVHD development (125). In a recent study, Kim et al. (132) showed that human MSCs, primed with IFNγ before infusion, displayed stronger suppression of GVHD *in vivo* in an indoleamine 2,3-dioxygenase (IDO)-dependent manner. On the other aspect, IFNγ production is essential for tumor eradication as well (133). GVT effect was diminished in the hosts receiving IFNγ-deficient donor cells as IFNγ was also shown to promote FasL-dependent GVT activity of CD8^+^ T cells (35). Furthermore, lack of IFNγ led to impaired Treg function and exacerbated GVHD (134). Among these studies, we note that IFNγ may function as a cytotoxic molecule as well as a proinflammatory cytokine. While both functions are involved in GVHD and GVT effect, a better mechanistic understanding of the INFγ signaling is still required for dissociating the GVT effect from GVHD.

### Tumor Necrosis Factor-Related Apoptosis-Inducing Ligand

Tumor necrosis factor (TNF)-related apoptosis-inducing ligand (TRAIL) belongs to TNF superfamily. TRAIL can induce target cell apoptosis though binding to death receptor (DR) 4 or 5 (135). TRAIL is upregulated after allogeneic stimulation and does not affect donor T cell proliferation and cytokine production. It has been reported that TRAIL contributes to optimal GVT effect since TRAIL^−/−^ donor T cells exhibit decreased anti-tumor activity (37). NK cell-derived TRAIL was also shown to kill acute lymphoblastic leukemia cells after HCT (136). A study using over-expression system revealed that TRAIL^+^ T cells induced less GVHD but augmented GVT effect (137), while another study reported that the level of soluble TRAIL in peripheral blood after allo-HCT was corelated with better prognosis with less GVHD (138), suggesting that TRAIL may be a feasible target for GVHD and GVT management.

## Conclusion and Perspective

The prevention and treatment of GVHD without impairing the GVT effect remains a major challenge for allo-HCT. Over the past decades, intriguing studies in the field of cytotoxic pathways open new avenues that can potentially diminish GVHD while largely preserving the GVT effect. It has been established that Fas/FasL, perforin/granzyme and cytokines are three major pathways contributing to T cell-mediated cytotoxicity in allo-HCT (Table 1). However, our understanding of these complicated pathways remains limited. There is still a barrier where current animal models cannot precisely mirror the clinical situation, leading to compounding discrepancies that hinder the translation into clinical practice. We anticipate that the improved insights of the cytotoxic pathways coupled with advanced technologies targeting these pathways will in the near future promote translation of preclinical discoveries into clinical implementation in GVHD management (139). New therapies, such as targeting GzmB, may emerge to overcome this devastating complication.

**Table 1:** Contribution of different cytotoxic pathways in allo-HCT.

**Cytotoxic pathway**	**Influence on GVHD**	**Influence on GVT**	**Target organs**
Fas/FasL	Contributes to both CD4^+^ and CD8^+^ T cell-mediated GVHD (46–54, 60, 67, 68). FasL in NK cells inhibits GVHD (72–74).	Controversial; seems more important for CD4^+^ T cell-mediated GVT (28, 35, 60, 85, 86).	Damage skin, liver, thymus, HSC, controversial for GI (75–84).
Perforin	Involved in CD8^+^ T cell-mediated GVHD (52, 68) Perforin in NK cells inhibits GVHD (74).	Critical for CD8^+^ T cell-mediated GVT (38, 97).	Not defined (31).
GzmB	GzmB is involved in CD8^+^ T cell-mediated GVHD (107). GzmB decreases CD4^+^ T cell-induced GVHD (111). GzmB does not affect natural Treg cell mediated suppression of GVHD (103).	GzmB damages CD8^+^ T cell-mediated GVT (107). GzmB contributes to optimal GVT induced by CD4^+^ T cells (104). GzmB does not affect natural Treg cell mediated suppression of GVT (103).	Not defined (31, 105, 107).
GzmA	GzmA is required for Treg-mediated suppression of GVHD (105, 106).	No report	Protects GI GVHD (105).
IFNγ	Controversial; Can be either protective against GVHD (126, 129–132), or dispensable for GVHD (127, 128). IFNγ increases Treg-mediated suppression GVHD (134).	IFNγ is critical for GVT effect (35, 36, 133).	No report
TNFα	TNFα is associated with GVHD development (112, 119).	No report	Damages skin, liver, GI (122).
TRAIL	TRAIL in T cells decreases GVHD (137). Soluble TRAIL prevents GVHD (138).	TRAIL is required for GVT effect (37, 136, 137).	No report

## Author Contributions

WD searched literature and wrote the manuscript. XC searched literature and wrote the manuscript.

### Conflict of Interest Statement

The authors declare that the research was conducted in the absence of any commercial or financial relationships that could be construed as a potential conflict of interest.

## Abbreviations

Allo-HCT: allogeneic hematopoietic cell transplantation
GVHD: graft-vs.-host disease
GVT: graft-vs.-tumor effect
TCD: T cell depletion
MHC: major histocompatibility complex
TCR: T cell receptor
FasL: Fas ligand
GzmA: granzyme A
GzmB: granzyme B
TNF-α: tumor necrosis factor alpha
IFN-γ: interferon gamma
TRAIL: tumor necrosis factor (TNF)-related apoptosis-inducing ligand.
